# Exploring the factor structure of the Health of the Nation Outcomes Scale (HoNOS) in a sample of patients with schizophrenia, schizotypal and delusional disorders

**DOI:** 10.1186/s12955-017-0701-1

**Published:** 2017-07-14

**Authors:** Wayne Smith, Silia Vitoratou, Paul McCrone, Anita Patel

**Affiliations:** 1 Health Service & Population Research Department, Institute of Psychiatry, Psychology and Neuroscience, King’s College London, London, UK; 2 Psychometrics and Measurement Lab, Biostatistics Department, Institute of Psychiatry, Psychology and Neuroscience, King’s College London, London, UK; 3 Centre for Primary Care and Public Health, Bart’s and the London School of Medicine and Dentistry, Queen Mary University of London, London, UK

**Keywords:** HoNOS, Factor analysis, Bifactor analysis, Bayesian, Factor structure

## Abstract

**Background:**

The Health of the Nation Outcomes Scale (HoNOS) is recommended for use by the English National Service Framework for Mental Health and by the working group on outcome indicators for severe mental illnesses to the Department of Health. It was developed to measure the health and social functioning of people with severe mental illness. Since the development of the HoNOS many have debated its latent structure. This paper examines the latent structure of the HoNOS using current factor analysis techniques.

**Method:**

HoNOS data for 12,910 patients with ICD10 diagnoses F20 to F29 at a UK National Health Service Mental Health Trust were analysed using exploratory, confirmatory and bifactor analysis for categorical data. The fit of models was assessed using relative and absolute fit indices.

**Results:**

Exploratory followed by confirmatory factor analysis identified a four factor solution which fit the data better than existing models. The corresponding bifactor factor solution identified three robust factors and one weak factor after accounting for a general factor. The factor loadings on the general factor were not appreciably different when compared to a unidimensional factor solution indicating the existence of a common trait.

**Conclusion:**

Existing models proposed in the literature did not fit well in our data. Factor analysis identified a new four factor solution. These factors showed clinical relevance according to published literature. The bifactor model demonstrated that there is not much loss of information when the HoNOS is used as a unidimensional construct. Further studies should explore this structure in larger samples and in alternative sample populations. A bifactor approach may have implications for how the HoNOS is used in practice, since there is ongoing debate on whether HoNOS item scores should be aggregated for interpretation.

## Background

The Health of the Nation Outcomes Scale (HoNOS) was developed to measure the health and social functioning of people with severe mental illness [1]. It is a recommended for use by the English National Service Framework for Mental Health and by the Department of Health and forms part of the English mental health minimum dataset (MHMDS) [2], a mandatory dataset for the National Health Service (NHS) funded care, including independent sector providers. It was initially developed by Wing et al. [3] and consists of 12 items measuring four subscales: behaviour, impairment, symptoms and social functioning. Each item is scored on a five-point Likert scale ranging from 0 (no problems) to 4 (severe to very severe problems) yielding a total score of 0 to 48. Completion of the HoNOS at two time points allows for the assessment of patient outcomes. A complete description of the items can be found in the HoNOS glossary [4].

Since its development many have debated the latent structure of the HoNOS [3, 5–17] and therefore this brings into question whether the instrument should be used as a total score outcome measure or scored according to appropriate subscales.

This paper examines the factor structure of the HoNOS using item factor analysis (that is factor analysis for categorical data). The aim is to determine whether the HoNOS has a multidimensional structure as outlined originally by its developers, whether alternative factor structures proposed in the literature may be more appropriate or, if necessary, to derive a new, more suitable factor structure.

## Methods

### Participants

For this study the data consisted of patients with an International Classification of Disease (ICD-10) diagnosis F20 to F29 [18]. An F20 to F29 diagnosis includes schizophrenia, schizotypal and delusional disorders, and other non-mood psychotic disorders such as psychosis in the absence of depression or bipolar disorder. The data represented a first HoNOS assessment during an admission for each patient and was extracted as part of a larger study aimed to develop a reduced health state classification system from the HoNOS which can be used in economic evaluations for patients with severe mental illness. Both HoNOS data and respective demographics for each patient were extracted from the Clinical Record Interactive Search (CRIS) database at the National Institute of Health Research Biomedical Research Centre (NIHR BRC), South London and Maudsley NHS (SLaM) Trust in the UK. The CRIS database is an electronic database of anonymised mental health data. The responses of 12,910 individuals were available for the analyses. This represented both inpatients and outpatients attending from 2010 to 2013. The statistics of the patient characteristics are presented in the results section.

### Assessing the latent structure

#### Item factor analysis

Common factor analysis for metrical data is often used in ordinal data [19]. However common factor analysis assumes that outcomes have symmetrical distributions. This assumption may be violated with Likert scale data in health outcome measures (as is the case with the HoNOS) [20]. The responses in all twelve items of the HoNOS were severely skewed to the right (indicating that the data consisted of a group of patients who were assessed as largely well on these items). As these asymmetries may introduce bias in the estimation of the parameters and their standard errors under the factor analysis model for metrical items, item factor analysis for categorical data was used [21].

Factor analysis for categorical data (often referred to as item factor analysis or latent trait model) via the weighted least squares estimator (WLSMV) [22] was implemented using Mplus [23]. For the complete assessment of the latent structure of the new measure we used exploratory item factor analysis (EFA) and confirmatory item factor analysis (CFA). The dimensionality of the scale was then further explored using the bifactor model [24]. The methods implemented are briefly described below.

#### Exploratory Factor Analysis (EFA)

EFA assesses the underlying structure of a set of variables with no a priori expectation on how the variables form factors. The model identifies the number of underlying latent variables (also called factors) and how each item loads onto these factors. Under this model framework, all items have some loading to each latent factor. A Geomin rotation [25] was used and salient loadings (that is, loadings at least as large as 0.3) are presented in bold to ease the interpretation of the latent factors. The rotation may reduce the number of cross-loading items, that is, items with large loadings (> 0.3) in more than one factor [26]. These items make the factor structure more complicated.

Parallel analysis using polychoric correlations [27] was used to identify the maximum number of factors to retain, along with the goodness of fit statistics.

#### Confirmatory Factor Analysis (CFA)

In CFA the factorial structure is pre-defined, that is the number of factors and how the items are assigned to each factor are imposed to the model by the user. CFA was applied here to test the fit of the factor structures which have been previously proposed in the literature, as well as those driven by the current EFA results. CFA models with and without cross-loading items are explored.

Methodologically, EFA and CFA models cannot be employed in the same data. For that reason, the sample was randomly divided into two samples; the first sub-sample (Sample 1) was used in EFA and the second one (Sample 2) in CFA.

#### Bifactor factor analysis

Bifactor modelling offers an appealing solution to conflicting evidence of unidimensionality and multidimensionality in outcome measures. It can inform us whether there is enough evidence for outcome measures to be used as a multidimensional structure, a unidimensional measure or both [28]. This is especially important in psychological constructs since they can have complex structures. In these cases item analysis often reveals the presence of a single overarching dimension but also uncovers unique clusters of items [29].

In the bifactor model all indicators are modelled to load on a single general latent factor. But item covariance can also be caused by competing specific factors which are uncorrelated with each other and with the general factor. Typically, each item can load on the general factor and only one specific factor [30] and each specific factor consists of three or more items which form independent clusters [28].

This is a restricted bifactor model which assumes that there are no cross-loadings. In the presence of cross-loadings (as is the case here), Reise et al. [31] suggests that these cross-loadings can result in biased estimations of the factor loadings. However, the restricted bifactor model can still be used to determine the general pattern of trivial and non-trivial loadings. The recent development of the bifactor Bayesian structural equation modelling (Bifactor BSEM) allows bifactor modelling in the presence of cross-loadings on the specific factors [32]. This was used to further corroborate the findings from the standard bifactor approach. The bifactor model was fitted here by augmenting the best model with the general factor.

Reise et al. [24] proposed that bifactor models could be used for exploring dimensionality using the following approach:

- Assess whether an outcome measure can be used as a unidimensional scale. If the factor loadings on the general factor in a bifactor solution remains comparable to the loadings in a unidimensional solution, then this indicates that no major loss in information is present when the scale is used as unidimensional.
- Assess whether the multidimensional factors are justified. If the factor loadings on the general factor are higher than the ones on the specific factor and, the factor loading on specific factors of the bifactor model are diminished compared to the multidimensional factor structure then this indicates a weak factor. In this case the specific factor has little influence after controlling for the general factor.

Reise’s approach was used here to further assess the dimensionality of the HoNOS.

#### Assessing model fit

Measures of both absolute and relative fit were used, namely the relative chi-square (relative χ^2^: values close to 2 indicate close fit; [33]), the Root Mean Square Error of Approximation (RMSEA; values less than 0.8 are required for adequate fit [34]), the Comparative Fit Index (CFI; values higher than 0.9 are required for close fit [35]), and the Standardised Root Mean square Residual (SRMR; values less than 0.8 indicate acceptable fit [36]). Data analyses were conducted in Mplus [23]. For EFA, parallel analysis (PA), [37] with principal component extraction of polychoric correlations were used identify the number of factors to extract. This was cited as the best approach for skewed, ordinal data with a small number of factors [38].

#### Reliability

In CFA models Cronbach’s alpha is presented for each factor as is customary. Cronbach’s alpha (α) determines the reliability of each set of items in a factor. Cronbach’s alpha is a function of the number of items in a scale so fewer items will result in a smaller coefficient [39]. For the bifactor model coefficient omega (ω) was used to estimate the reliability of the general and specific factors [40]. Following this hierarchical omega coefficient (ω_H_) was used to estimate the reliability of the general factor and, omega subscale (ω_S_) used to estimate the reliability of the specific factors by controlling for the general factor.

## Results

### Demographics

Table 1 outlines the demographic characteristics of the entire sample. Independent samples t-test showed that females were on average 6 years older than males (females mean age: 45, sd = 15 years, males mean age: 39, sd = 13 years *t* = −23.985, df = 12,908, *p* ≤ 0.001).

**Table 1:** Descriptive indices of sample

		Age	Ethnicity
Gender	Male 7631 (59%)	Mean (SD): 39 (13)	Black: 3953 (52%)
		Max: 92	White: 3130 (41%)
		Min: 18	Other: 548 (7%)
	Female 5279 (41%)	Mean (SD): 45 (16)	Black: 2834 (54%)
		Max: 97	White: 2060 (39%)
		Min: 18	Other: 385 (7%)
	Overall 12,910	Mean (SD): 42 (15)	
		Max: 97	
		Min: 18	

There were significant differences in the mean ages between ethnic groups (F_(2, 12,907)_ = 171.59, *p* ≤ 0.001). Bonferroni post hoc pairwise comparisons (adjusted for multiple comparisons) showed that on average “white” individuals were 4.9 years older than “black” or “other” ethnicity group (“white” mean age = 45, sd = 15 years, “black”: mean age = 40, sd = 13 years, “other” mean age = 40, sd = 14 years; *p* ≤ 0.001). However, there was no significant difference between the “black” and “other” ethnicity groups (*p* ≥ 0.999).

### EFA

Table 2 reports on the goodness of fit indices for all EFA models from the unidimensional to the five factors solution. The fit of the one factor model was not adequate, suggesting that the scale is not unidimensional. The fit improved at the three factor solution and close fit was achieved at the four factor solution.

**Table 2:** Goodness of fit indices for the EFA models (one to five factors solutions)

Goodness of fit indices	Model				
	1 Factor model	2 Factor model	3 Factor model	4 Factor model	5 Factor model
Relative χ^2^	65.36	37.53	27.41	13.33	5.38
CFI	0.69	0.86	0.92	0.97	0.99
RMSEA (90% CI) Probability RMSEA <= 0.05	0.102 (0.099, 0.105) *p* < 0.001	0.077 (0.074, 0.080) *p* < 0.001	0.065 (0.062, 0.069) *p* < 0.001	0.045 (0.040, 0.049) *p* = 0.980	0.027 (0.021, 0.032) *p* > 0.999
SRMR	0.09	0.06	0.05	0.03	0.01

**Fig. 1:**
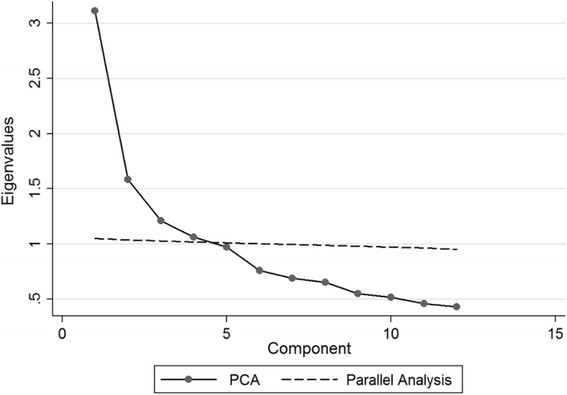
Parallel analysis extraction using polychoric correlations

As the number of factors increases the fit always improves. In terms of the axiom of parsimony, one should stop extracting factors once close fit is achieved. A five factor solution in the current data would result in over-extraction. This is further indicated by the fact that the five factor solution consists of a two item factor, more cross-loading items, and some of the item factor loadings on factors become smaller. Finally, PA also suggested four factors, as demonstrated in the scree plot (see Fig. 1). Therefore, we conclude that EFA suggests a four factor solution in line with the results of the PA.

Table 3 presents the pattern matrix (Geomin rotation) for the four factor EFA solution. In the final solution there were two items with cross-loadings. Item 8 loaded saliently on the first and second factors, whilst item 10 loaded on the third and fourth factors. The EFA driven models, with and without cross-loadings are tested via CFA in the next section, along with the models proposed previously in the literature.

**Table 3:** EFA factor loadings under a Geomin rotation for the four factor solution

Item	Extracted factor			
	1	2	3	4
I01 – Overactive, aggressive, disruptive or agitated behaviour	**0.78**	−0.09	0.00	−0.01
I02 – Non-accidental self-injury	0.25	**0.47**	−0.07	−0.03
I03 – Problems with drugs or alcohol	**0.32**	0.01	−0.19	0.15
I04 – Cognitive problems	0.13	0.08	**0.45**	0.04
I05 – Physical illness or disability problems	−0.13	−0.02	**0.47**	−0.04
I06 – Problems associated with hallucination and delusions	**0.53**	0.06	0.04	−0.02
I07 – Problems with depression	−0.03	**0.91**	0.01	0.01
I08 – Other mental health problems	**0.33**	**0.30**	0.07	0.03
I09 – Problems with relationships	0.25	0.06	0.05	**0.38**
I10 – Problems with activities of daily living	0.02	−0.01	**0.54**	**0.45**
I11 – Problems with living conditions	−0.03	−0.02	−0.05	**0.63**
I12 – Problems with occupation and activity	−0.02	0.00	0.02	**0.67**

Salient factor loadings (>0.3) are presented in bold

### CFA

Table 4 presents the factor loadings and Cronbach’s alpha for each factor of the ten models which were evaluated with CFA using the second split half sample. The first three were based on the EFA results. In particular, M1 stands for the four factor solution where each item was assigned to the factor where it loads the highest (Table 3). M2 is slightly augmented by placing the cross-loading of item 8 to the second factor. M3 is further augmented by allowing item 10 to load also on the fourth factor as EFA indicated a substantial cross-loading. Models M4 to M12 correspond to models previously suggested in the literature (see Table 4 for a complete description and references).

**Table 4:** Structure and CFA goodness of fit, for the EFA-based models and the models previously proposed in the literature

Latent Factors		Factor 1 (alpha)	Factor 2 (alpha)	Factor 3 (alpha)	Factor 4 (alpha)	Stand-alone item(s)	Relative χ^2^	RMSEA	CFI
EFA 1	(M1)	1,3,6,8 (0.515)	2,7 (0.456)	4,5,10 (0.466)	9,11,12 (0.542)	−	33.34	0.065 (0.062, 0.068)	0.93
EFA 2	(M2)	1,3,6,8 (0.515)	2,7,8 (0.487)	4,5,10 (0.466)	9,11,12 (0.542)	−	27.04	0.062 (0.060, 0.065)	0.93
EFA 3	(M3)	1,3,6,8 (0.515)	2,7,8 (0.487)	4,5,10 (0.466)	9,10,11,12 (0.635)	−	24.72	0.059 (0.056, 0.062)	0.93
Wing [3]	(M4)	1,2,3 (0.360)	4,5 (0.252)	6,7,8 (0.496)	9,10,11,12 (0.635)	−	50.25	0.072 (0.069, 0.075)	0.90
McClelland [7]	(M5)	1,3,4,6, 9–12 (0.641)	2,7 (0.456)	−	−	5,8	35.7	0.086 (0.083, 0.088)	0.85
Newnham [10]	(M6)	1,2,3,9 (0.451)	4,5,6,10 (0.437)	7,8 (0.443)	11,12 (0.562)	−	51.77	0.087 (0.084, 0.090)	0.85
Trauer [16]	(M7)	1,3 (0.338)	4,5 (0.252)	2,7,8,9 (0.499)	9,10,11,12 (0.635)	6	23.98	0.058 (0.055, 0.061)	0.94
Speak et al. [12]	(M8)	1,6 (0.451)	2,7,8 (0.487)	4,5,10,12 (0.518)	3,9,11,12 (0.485)	−	30.56	0.066 (0.063, 0.069)	0.91
Speak & Muncer [13]	(M9)	9,10,11,12 (0.635)	−	−	−	−	30.44	0.066 (0.052, 0.081)	0.99
Lovaglio [41]	(M10)	4,6,9–12 (0.619)	−	−	−	−	47.84	0.083 (0.077, 0.090)	0.95
Muncer [8]	(M11)	2,7,8 (0.487)	10,11,12 (0.605)	−	−	−	14.28	0.044 (0.035, 0.054)	0.98
Muncer [9]	(M12)	2,7,8,9 (0.499)	9,10,11,12 (0.631)				17.50	0.050 (0.042, 0.068)	0.98

Among the models previously suggested in the literature, some utilise all 12 items (M4, M5, M6, M7 and M8) and others use a reduced number of indicators (M9, M10, M11 and M12). Among those which contain all 12 items, the model M7 had the closest fit to the data, according to the goodness of fit indices (see Table 4). However, this model states that item 6 stands as an exogenous observed variable. That is, item 6 is not an indicator of a latent factor but rather directly measures what is indicated (i.e. item six – *problems associated with hallucination and delusions*).

Regarding the reduced items models, M11 showed the best fit to the data. However, the model represents a reduced measure consisting of two factors each with three items. M9 also showed good fit. This model retains only the social items, as defined by Wing et al. [3] *(problems with relationships, activities of daily living, problems with living conditions and problems with occupation and activities)* to from one factor. However, retaining only four items as in M9 may reduce the clinical usefulness of the HoNOS since other items (such as item 1: *over-activeness, aggression, disruptive or agitated behaviour* and item 7: *problems with depressed mood*) which evaluate behaviours and mental state are excluded. This is also the case for Lovaglio’s model (M10; [41]), who in order to develop a unidimensional scale reduced the scale to 6 items.

Since Trauer’s model [16] has a stand-alone item and Muncer’s [9] model represented a much reduced HNOS version we decided to exclude both of these as options for best fitting models. The most current 12-item model M8 (Speak et al. [12]) did not fit these data as well as M3 (the best fitting model with 12 items developed through EFA/CFA). M3 had at least three items in each factor and produced better fit indices compared with M8.

In addition, α coefficients were approximately 0.5 for the first three factors of the M3 model and 0.6 for the fourth factor showing good reliability across all factors compared to other models. The α coefficients for the first and second factors of Trauer’s model [16] were approximately 0.3 indicating low reliability of these two factors. Both of these factors consisted of two items only. The coefficient of the fourth factor of Speak et al.’s model [12] was smaller than M3’s (approximately 0.5 versus 0.6 respectively) even though they contained the same number of items. This was also an indication that the fourth factor of M3 was more reliable.

### Bifactor model

The bifactor BSEM methodology was used to test the dimensionality of the model with the best fit (M3). The factor loadings for the unidimensional, bifactor BSEM, and four factor solutions can be found in Table 5. First, the loadings of the general factor of the bifactor model were compared to the ones of the only factor in the unidimensional solution. Item by item, the loadings were not appreciably different between the two models indicating that HoNOS can be used as a unidimensional scale.

**Table 5:** Comparison of factor loadings between the unidimensional (Uni), Bifactor BSEM and multidimensional CFA model

	Uni	Bifactor BSEM ^a^ (Loading of Standard bifactor model)					Four factor solution			
Item (I)	Factor	General	S1	S2	S3	S4	F1	F2	F3	F4
I01 – Overactive, aggressive, disruptive, agitated	0.55	1.15* (0.61)	0.30 (0.14)				0.67			
I02 – Non-accidental self-injury	0.45	0.66* (0.43)		0.86* (0.39)				0.69		
I03 – Problems with drugs or alcohol	0.23	0.28* (0.29)	0.36 (0.12)				0.26			
I04 – Cognitive problems	0.44	0.43* (0.42)			0.44* (0.38)				0.74	
I05 – Physical illness or disability problems	0.21	0.10* (0.12)			0.60*(0.45)				0.34	
I06 – Problems with hallucination & delusions	0.52	0.76* (0.56)	0.14 (0.71)				0.65			
I07 – Problems with depression	0.43	0.48* (0.39)		1.07* (0.80)				0.70		
I08 – Other mental health problems	0.46	0.55* (0.45)	−0.13 (0.12)	0.32* (0.21)			0.32	0.30		
I09 – Problems with relationships	0.55	0.54* (0.52)				0.38* (0.26)				0.61
I10 – Problems with activities of daily living	0.64	0.62* (0.43)			0.81*(0.53)	0.73* (0.51)			0.31	0.52
I11 – Problems with living conditions	0.51	0.33* (0.30)				0.82* (0.59)				0.64
I12 – Problems with occupation and activity	0.55	0.38* (0.35)				0.84* (0.59)				0.60
		ω = 0.827	ω = 0.665	ω = 0.689	ω = 0.616	ω = 0.784				
		ω_H_ = 0.603	ω_S_ = 0.163	ω_S_ = 0.378	ω_S_ = 0.409	ω_S_ = 0.600				

Fit of unidimensional model: Relative Chi = 60, CFI = 0.83, RMSEA = 0.09

Fit of multidimensional: Relative Chi = 25, CFI = 0.93, RMSEA = 0.059

(*) Significant loadings for the Bifactor BSEM (*p* < 0.05)

(ω) Omega, (ω_H_) Omega hierarchical, (ω_S_) Omega subscale

^a^ A common bi-factor model was also used and the fit was: Relative Chi = 14, CFI = 0.97, RMSEA = 0.044

Second, we compared the loadings of the items to the specific factors of the bifactor BSEM (S1, S2, S3 and S4) to the corresponding ones in the factors (F1, F2, F3 and F4) of the four factor solution. Overall the factor loadings on the three specific factors (S2, S3 and S4) were high and comparable to the corresponding ones in F2, F3 and F4. Overall, these factors formed stable solutions with high factor loadings.

Regarding the first factor (S1), when the general factor was added in the bifactor model the loadings on three of the items in S1 (items 1, 3 and 8*)* dropped appreciably. As outlined in the Methods section this indicates a weak factor with little influence after accounting for the general factor. The result of the Bifactor BSEM also shows that all factor loadings were significant apart from items 3, 6 and 8 in S1 of the bifactor BSEM model. This also corroborates the findings of three strong factors with high factor loadings on each factor and a weak factor with low factor loadings. Reliability indices (ω, ω_H,_ and ω_S_) for the standard bifactor model are also presented in Table 5. As expected ω values were lower than ω_H_ and ω_S_ values. After accounting for the general factor ω_S_ on specific factor S1 of the bifactor model dropped considerably compared to S2-S4.

## Discussion

Regarding the models previously proposed in the literature, in our data Wing’s original model [3] did not fit well to our data. From the remaining models in the literature which consisted of all twelve items and did not have a stand-alone item in their structure, Speak et al. [12] showed the best fit compared to the other models. However, we were able to find a four factor model (M3) which had better fit to these data than all other models. Trauer [16] solution had almost the same fit to our data with model M3, but in its case an exogenous variable is included in the model.

There are two cross-loading items in our proposed four factor solution. Item 8 loaded on the first and second factor and item 10 loaded on the third and fourth factor. There are established relationships between item 8 *(other mental health problems)* and the other items within this factor; item 3 (*drug and alcohol use*), item 6 (*experiencing hallucinations and delusions*), and item 1 (*overactive aggressive or disruptive behaviours*). Coexisting mental health and drug and alcohol problems are common in clinical practice [42] and drugs and alcohol can cause psychiatric symptoms and mimic psychiatric disorders with links between depression cocaine use and schizophrenia and polydrug addiction [43]. In addition individuals with mental illness can also have multiple diagnoses (diagnostic comorbidities) [44] and therefore it is plausible for item 8 with such a broad description to also fit well with item 2 (*non-accidental self-injury*) and item 7 (*problems with depression*). Therefore, we consider the cross-loadings justified and in line with the latent constructs.

Regarding item 10 (*activities of daily living* or ADLs), this fits well within both the third and fourth factor. Item 10 (ADLs) forms a plausible factor with item 4 (cognitive problems) and item 5 (physical problems). Network and Stoppe [45] state that a decline in physical autonomy and functional impairment in dementia is related to cognitive decline and discuss the loss of ability to perform ADLs. Most cognitive or physical impairment will have an effect on one’s ability to perform routine tasks. Clinically it is also plausible for item 10 (ADLs) to affect other social aspects of life such as relationships (item 9), accommodation (item 11) and problems with occupation and activities (item 12).

The bifactor model confirmed three robust specific factors with good factor loadings even after accounting for the general factor and one weak specific factor which had poor loadings. It is possible that this factor might be a ‘bloated specific’ factor [46] where items emerge because of inflated covariance due to high item content overlap. We conclude that the general factor of the bifactor model reflects the common trait ‘Overall mental health or well-being’ and the four specific factors represented additional sources of common variance which may be due to item content [24]. The four specific factors were named as follows: ‘Cognitive or physical illness and personal care’ (items 4, 5 &10), ‘Social issues’ (9,10,11 & 12), ‘Emotional stability’ (items 2,7 & 8) and, ‘Addictions, behaviour and mental health diagnoses’ (items 1,3,6 & 8). Although the fourth factor may not be a reliable one due to poor factor loadings and a low omega subscale score.

We have referenced and discussed above evidence in the mental health literature to support grouping the items in the ‘Emotional stability’ and in the ‘Addictions’ category. In addition, there is evidence from earlier factor analyses which support the two remaining factors. The items in the ‘Social issues’ factor have been identified as early as Wing’s model [3] and again in Trauer’s [16] and Speak and Muncer’s model [13]. The items in ‘Emotional stability’ have also be proposed as a factor by Speak et al. [12].

One key limitation of this study is that we have used a sample of patients with ICD10 diagnoses of F20 to F29. As a result this factor structure is not one which has been proposed for all areas of use. It was identified solely to understand the structure within this group of patients so that we may apply additional psychometric techniques to form a reduced HoNOS classification system to be used in health economic evaluations for patients with severe mental illness. In a recent paper Speak and Muncer [15] applied confirmatory factor analysis for ordinal data to the HoNOS. They tested various factor structures in subgroups of mental health clusters and superclasses.

Another limitation of the study is with regards to the bifactor model. Cross-loading items may have had an effect on the factor loadings of both the general and specific factors for the standard bifactor model and therefore the omega estimates may not have been accurate. Bifactor BSEM is a relatively new approach and therefore we did not have the capability to explore the possibility of calculating reliability estimates for this approach.

## Conclusion

In this work we used EFA, CFA and a bifactor model to identify the factor structure of HoNOS in a sample of patients with ICD10 diagnoses F20 to F29. Our data did not support the factor structure proposed by the Wing et al. [3] original subscale structure. Trauer [16], Speak et al. [12], Speak & Muncer [13] showed good fit to the data. But, the model that uses all twelve items and all items as indicators of latent constructs, is M3.

EFA and CFA indicated that a four factor solution M3 is the most acceptable solution. Using the bifactor model we were also able to support the use of the HoNOS as a unidimensional scale. This bifactor model acknowledges the existence of both a general factor and coexisting specific factors with the first specific factor having little influence (low factor loadings) compared with the three remaining factors. All four factors showed clinical relevance according to published literature. Future studies should examine this structure and the bifactor approach in alternative subclasses of patients.

This study contributes to the research which aims to establish how the HoNOS ratings should be used to evaluate outcomes. It has implications for whether the ratings should be aggregated in practice. Deriving an appropriate factor structure will help to improve the sensitivity of the HoNOS with respect to changes in subscale scores. It is important to establish a consistent reliable approach to using the HoNOS ratings to be able to identify significant changes and efficiently assess patients’ needs. This study was based on a sample of a patients with severe mental illness. Further studies could apply this approach to a different patient population or a wider patient group.

## Abbreviations

ADLs: Activities of daily living
BSEM: Bayesian structural equation modelling
CFA: Confirmatory factor analysis
CFI: Comparative fit index
CRIS: Clinical record interactive search
EFA: Exploratory factor analysis
HoNOS: Health of the nation outcomes scale
ICD –10: International classification of disease
NHS: National Health Service
NIHR: BRC National Institute of Health Research Biomedical Research Centre
PA: Parallel analysis
RMSEA: Root mean square error of approximation
SLaM: South London and Maudsley
SRMR: Standardised root mean residual
UK: United Kingdom
WLSMV: Weighted least squares estimator
